# Appraisal of Laparoscopic Distal Pancreatectomy for Left-Sided Pancreatic Cancer: A Large Volume Cohort Study of 152 Consecutive Patients

**DOI:** 10.1371/journal.pone.0163266

**Published:** 2016-09-16

**Authors:** Sang Hyun Shin, Song Cheol Kim, Ki Byung Song, Dae Wook Hwang, Jae Hoon Lee, Kwang-Min Park, Young-Joo Lee

**Affiliations:** Division of Hepato-Biliary and Pancreatic Surgery, Department of Surgery, Asan Medical Center, University of Ulsan College of Medicine, Seoul, South Korea; University of Crete, GREECE

## Abstract

**Background:**

The aim of this study was to appraise the value of laparoscopic distal pancreatectomy (LDP) for left-sided pancreatic cancer based on a large volume cohort study.

**Methods:**

We reviewed data for all consecutive patients undergoing LDP for left-sided pancreatic cancer at Asan Medical Center (Seoul, Korea) between December 2006 and December 2014.

**Results:**

A total of 91 male and 61 female patients, with a median age of 62.7 years were included in this study. The median operative duration was 234 minutes. Pathological reports revealed the following: a median tumor size of 3.0 cm (range, 0.4–10.0), T stages (T1 in 7.9%, T2 in 5.3%, T3 in 86.8%, and no T4), the tumor differentiation (well differentiated in 16.4%, moderately differentiated in 75.4%, and poorly differentiated in 8.2%), and R0 resection in 126 patients (82.9%). After pancreatectomy, 96 patients (63.2%) received adjuvant chemotherapy, and the median time to chemotherapy was 30 days. The median length of hospital stay was 8 days (range, 5–31), and the median time to diet resumption was 1 day. Grade B or C postoperative pancreatic fistula occurred in 14 patients (9.2%) and grade II or III complications occurred in 27 (17.7%). The median overall survival was 43.0 months. A Cox proportional hazards model showed that tumor size, N1 stage, combined resection, and incompleteness of planned adjuvant chemotherapy affect patient survival.

**Conclusions:**

LDP for left-sided pancreatic cancer is reasonable within selected indications. An international consensus on laparoscopic surgery for pancreatic cancer would be desirable and timely.

## Introduction

In the current era of minimally invasive surgery, laparoscopic or robotic surgeries have become a new standard paradigm for the various procedures performed to treat benign tumors, malignant tumors, and even transplantation [1–5]. The laparoscopic procedure for managing pancreatic lesions has been standardized worldwide and its use has markedly increased since Gagner and colleagues [6] reported the first laparoscopic distal pancreatectomy (LDP) in 1996. The laparoscopic approach improves the visualization of retroperitoneal organs, and the absence of complicated anastomosis in distal pancreatectomy has led to LDP becoming the most commonly performed minimally invasive procedure in pancreatic surgery. Various previous studies have reported that laparoscopic pancreatectomy shows similar or better results than open pancreatectomy in terms of postoperative outcomes [7–11]. However, these procedures were mainly performed in benign or low-grade malignant diseases, and the consensus on laparoscopic pancreatectomy for malignant diseases remains to be established.

Our institute, which is a leading tertiary care hospital in South Korea, has extensive experiences with laparoscopic pancreatic surgery [10,12–15]. Our indications for laparoscopic pancreatectomy have been expanded to include more complicated diseases such as pancreatic cancer. We have already reported a comparative propensity score-matched analysis of laparoscopic versus open distal pancreatectomy (ODP) for left-sided ductal adenocarcinoma [14]. That study was one of the largest studies to use propensity score-matching to investigate the outcomes of laparoscopic surgery for left-sided pancreatic ductal adenocarcinoma. In that report, LDP showed comparable oncologic outcomes to ODP and was associated with a shorter length of hospital stay and an earlier return to diet than the matched ODP group.

In the present study, we analyzed the clinicopathological characteristics and postoperative outcomes of the largest series of patients who underwent LDP for resectable left-sided pancreatic cancer in a single center. The objectives were to introduce our experiences and to appraise the value of LDP for left-sided pancreatic cancer.

## Patients and Methods

### Patient Database

Between December 2006 and December 2014, 462 consecutive patients with left-sided pancreatic cancer underwent surgical resection at Asan Medical Center (Seoul, South Korea). Of the 462 patients, 169 (36.6%) underwent LDP. Their clinical, pathological, and surgical data were retrospectively reviewed using the electronic medical records of our institute. This study was approved by Asan Medical Center Institutional Review Board. Our institutional review board waived the need for written informed consent from the participants. Postoperative pancreatic fistulas (POPFs) and overall complications were assessed and graded based on the criteria of the International Study Group of Pancreatic Fistula (ISGPF) [16] and Clavien-Dindo complication classification [17], respectively. Resection margin status was categorized as R0 or R1 (pancreatic transection or retroperitoneal margins). If the closest safe resection margin was less than 1 mm, it was categorized as an R1 retroperitoneal margin.

Follow-up data were also obtained from the electronic medical records, and the duration of survival after surgery was measured from the time of surgery until death or the last visit to the outpatient department. All patients were preoperatively assessed using computed tomography (CT) and magnetic resonance cholangiopancreatography (MRCP). As a diagnostic strategy of our institute, most patients with PDAC preoperatively underwent 18F-fluorodeoxyglucose positron emission tomography (FDG-PET) for initial cancer staging to find hidden metastasis. Endoscopic ultrasonography (EUS) and/or EUS-guided fine-needle aspiration biopsy were sometimes performed for accurate diagnosis in selected patients. After surgical resection, all patients underwent CT to assess surgical complications, including POPF, on the 3rd or 4th postoperative day. As postoperative surveillance, CT was performed and CA 19–9 levels were examined every 3 months in the first 2 postoperative years and then every 6 months in the subsequent years in all patients. If necessary, FDG-PET, chest CT, and/or biopsy were also performed to confirm recurrence.

### Operative Procedure

First of all, current indications for LDP in our institute include the followings: (1) no distant metastasis, (2) no invasion to major vascular structures, (3) no involvement of adjacent organs, (4) no intraabdominal adhesion, and (5) no comorbidities precluding laparoscopic surgery. However, when unexpected involvement to adjacent organ is found during operation, we attempt laparoscopic en-bloc resection. Nowadays, there have been an increasing number of patients with locally advanced disease who undergo neoadjuvant therapy followed by surgical resection. These patients are candidates mainly for open surgery due to possibility of vascular reconstruction. When the patient is considered for LDP, the patient is informed about advantages and disadvantages of both ODP and LDP, and LDP is decided preoperatively when the patient agrees to receive a laparoscopic procedure.

To transect the pancreas safely, rotated endoscopic linear staplers of various sizes (staple height, 3.5 to 4.2 mm) were used, depending on the thickness or hardness of the pancreas. After transecting the pancreas in the neck, pancreatosplenectomy is performed in an antegrade manner. Based on the concept of radical antegrade modular pancreatosplenectomy (RAMPS) [18], we perform en-bloc resection of peripancreatic retroperitoneal tissues to ensure that there is no residual tumor. The dissection plane of the RAMPS procedure is aiming at exposing the superior mesenteric artery, left side of the aorta, renal vessels, renal parenchyma, and adrenal gland. However, it is sometimes time-consuming to secure all planes of the RAMPS, so we sometimes modify the concept of the RAMPS according to the location or depth of the tumor. We concentrate on securing the retroperitoneal radial margin of the tumor rather than uniformly exposing whole planes of RAMPS, especially when the tumor is located in the proximal body or far tail of the pancreas.

### Statistical Analysis

Categorical variables are expressed as number and percentage, and continuous variables are reported as median and range. The entire study cohort and those who underwent LDP for ductal adenocarcinoma between December 2006 and December 2014 were included in the analysis of the cumulative survival rates of the patient and the disease-free survival calculated using the Kaplan-Meier method. To estimate the factors affecting patient survival following LDP, the Cox proportional hazards model was performed. All statistical analyses were conducted using IBM SPSS version 18.0 (IBM SPSS).

## Results

### Patient Characteristics

Of a total of 169 patients who underwent LDP for left-sided pancreatic cancer during the study period, 7 had stage IV disease, 6 were lost to follow-up, and 4 underwent open conversion. Thus, the remaining 152 patients were included in this study. The reasons for open conversion were as follows: invasion of the common hepatic artery, invasion of the superior mesenteric vein, massive bleeding, and severe adhesions caused by previous colon operation. The demographic characteristics of all patients who underwent LDP are presented in Table 1. There were 91 women and 61 men, with a median age at diagnosis of 62.7 years (range, 30–88). Most patients were included in ASA score grade I or II because patients with poor general condition (ASA score grade IV or V) were not candidates for laparoscopic surgery. Regarding comorbidity, 51 patients had preoperative diabetes mellitus, 71 had cardiovascular disease, and 6 had pulmonary disease. The median preoperative body mass index was 24.0 kg/m^2^ (range, 16.6–32.2).

**Table 1:** Demographic characteristics of all 152 patients who underwent LDP.

Characteristics	N or median	% or range
**Sex**		
Female	91	59.9
Male	61	40.1
**Age, year**	62.7	30–88
**ASA score**		
Grade I	38	25.0
Grade II	105	69.1
Grade III	9	5.9
Grade IV-V	0	0.0
**Comorbidity**		
DM	51	
Cardiovascular	71	
Pulmonary	6	
**BMI, kg/m**^**2**^	24.0	16.6–32.2

DM, diabetes mellitus; BMI, body mass index

### Operative Features

The operative features of all 152 patients are listed in Table 2. The median operative duration was 234 minutes (range, 121–475). During LDP, 13 patients (8.6%) underwent laparoscopically combined resection of directly invaded organs to secure margins: wedge resection of the stomach in 7 patients, left hemicolectomy in 3, sleeve resection of the duodenal 4th portion in 2, and resection and anastomosis of the proximal jejunum in 1. Of the entire cohort, 4 patients (2.6%) received intraoperative blood transfusion.

**Table 2:** Operative features.

Features	N or median	% or range
**Operative duration, min**	234	121–475
**Combined resection, laparoscopically**		
No	139	91.4
Yes	13	8.6
Wedge resection of stomach	7	
Left hemicolectomy	3	
Sleeve resection of duodenal 4th portion	2	
Resection and anastomosis of proximal jejunum	1	
**Intraoperative transfusion**		
No	148	97.4
Yes	4	2.6

### Pathological Features

Pathological features are described in Table 3. Histological diagnoses of the resected left-sided pancreatic cancers, with a median size of 3.0 cm (range, 0.4–10.0), were of 130 ductal adenocarcinomas (85.5%), 18 invasive intraductal papillary mucinous neoplasms (11.8%), and 4 mucinous cystadenocarcinomas (2.6%). Most patients (86.8%) had T3 cancer. The median number of harvested lymph nodes (LNs) was 11 (range, 0–42), and 65 patients (42.8%) had a positive LN metastasis (N1 stage). R0 resection was achieved in 126 patients (82.9%) and R1 resection in 26 (17.1%). In addition, there was microscopic involvement of the retroperitoneal margin in 25 patients (16.4%) and of the pancreatic transection margin in 1 case (0.7%). Lymphovascular and perineural invasion were present in 45 (29.6%) and 105 (69.1%) patients, respectively.

**Table 3:** Pathological features (n = 152).

Features	N or median	% or range
**Histologic diagnosis**		
Ductal adenocarcinoma	130	85.5
Invasive intraductal papillary mucinous neoplasm	18	11.8
Mucinous cystadenocarcinoma	4	2.6
**Tumor size, cm**	3.0	0.4–10.0
**T stage**		
T1	12	7.9
T2	8	5.3
T3	132	86.8
T4	0	0.0
**N stage**		
N0	87	57.2
N1	65	42.8
**Number of harvested LN**	11	0–42
**Differentiation**		
Well	22	16.4
Moderate	101	75.4
Poor	11	8.2
NA	18	-
**Resection margin status**		
Negative (R0)	126	82.9
Pancreatic transection margin (R1)	1	0.7
Retroperitoneal margin (R1) ^a^	25	16.4
**Lymphovascular invasion**		
Absent	107	70.4
Present	45	29.6
**Perineural invasion**		
Absent	47	30.9
Present	105	69.1

LN, lymph node; NA, not available

^a^ In 11 patients, the cancer didn’t penetrate the tangential margin, but their safety margin was less than 1mm and they were categorized as R1.

### Postoperative Outcomes

Postoperative outcomes are indicated in Table 4. After pancreatectomy, 96 patients (63.2%) received adjuvant chemotherapy, and their median time to chemotherapy was 30 days (range, 19–70). Of these 96 patients, 72 (75%) completed all planned adjuvant chemotherapy cycles.

**Table 4:** Postoperative outcomes.

Outcome variables	N or median	% or range
**Adjuvant chemotherapy**		
No	56	36.8
Yes ^a^	96	63.2
**Time to chemotherapy, day**	30	19–70
**Length of hospital stay, day**	8	5–31
**Time to restarting diet (water), day**	1	1–6
**Time to restarting diet (liquid diet), day**	2	2–8
**Pancreatic fistula (ISGPF grade)**		
None	104	68.4
Grade A	34	22.4
Grade B	9	5.9
Grade C	5	3.3
**Complication Classification (Clavien-Dindo)**		
None	91	59.9
Grade I	34	22.4
Grade II	18	11.8
Grade III	9	5.9
Grade IV	0	0.0
Grade V	0	0.0

ISGPF, international study group of pancreatic fistula

^a^ Of 96 patients, 72 (75%) completed full cycles of adjuvant chemotherapy.

The median length of hospital stay was 8 days (range, 5–31), and the median time to water intake and a liquid diet were 1 and 2 days, respectively. Overall, POPF occurred in 48 patients (31.6%), and clinically relevant POPF (ISGPF grade B or C) was observed in 14 patients (9.2%). Complications of Clavien-Dindo grade II or higher occurred in 27 patients (17.7%). There was no in-hospital or 30-day mortality.

### Survival Analysis

**Fig 1:**
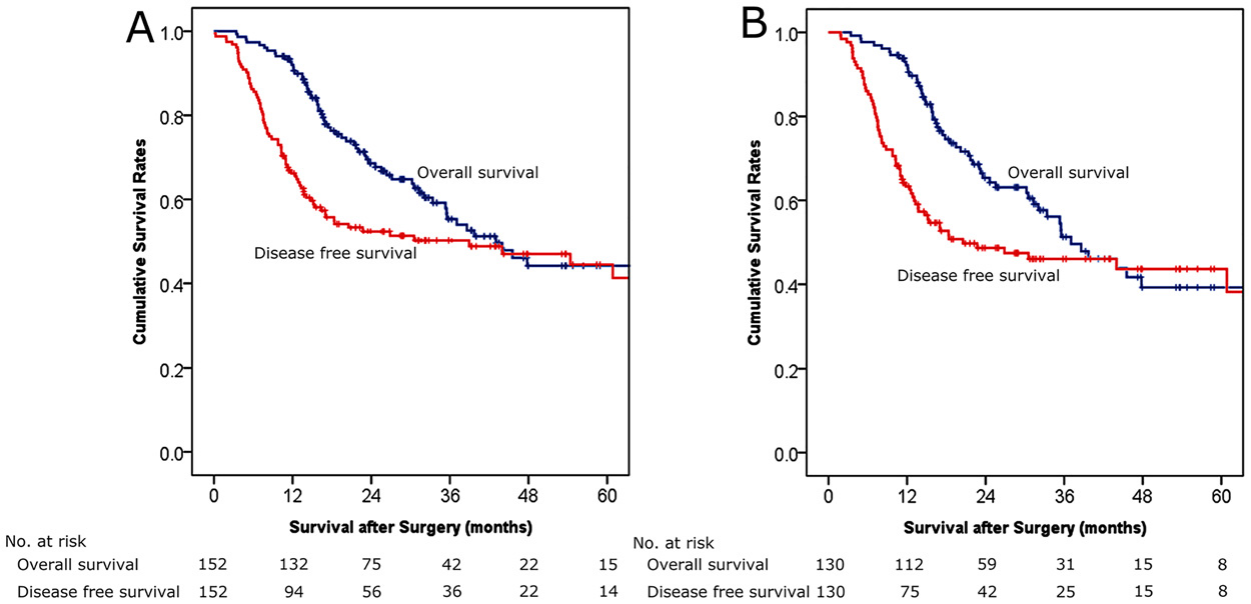
Kaplan-Meier curves of patient survival and disease-free survival. (A) In the entire cohort (n = 152), the median patient survival was 43.0 months, and the 1-, 3-, and 5-year survival rates were 92.0%, 55.3%, and 44.7%, respectively. The 1-, 3, and 5-year disease-free survival rates were 66.7%, 50.8%, and 45.0%, respectively. (B) In the pancreatic ductal adenocarcinoma cases (n = 130), the median survival was 37.0 months, and the 1-, 3-, and 5-year survival rates were 92.2%, 51.3%, and 39.3%, respectively. The 1-, 3, and 5-year disease-free survival rates were 63.3%, 46.0%, and 43.6%, respectively.

In the entire cohort (n = 152; Fig 1A), the median survival was 43.0 months and the 1-, 3-, and 5-year patient survival rates were 92.0%, 55.3%, and 44.7%, respectively. The 1-, 3-, and 5-year disease-free survival rates were 66.7%, 50.8%, and 45.0%, respectively. When patients who underwent LDP for ductal adenocarcinoma (n = 130) were included in the analysis of the patient overall and disease-free survival outcomes (Fig 1B), the median survival was 37.0 months and the 5-year patient survival and disease-free survival rates were 39.3% and 43.6%, respectively.

To estimate the factors affecting patient survival following LDP, we performed multivariable analysis using the Cox proportional hazards model. In this analysis, several independent survival factors were identified (Table 5). In the entire cohort, younger patients had relatively poorer survival (hazard ratio [HR] = 0.93). Larger tumor size (HR = 1.31), presence of lymph node metastasis (HR = 3.11), and combined resection of invaded adjacent organs (HR = 4.50) were included as dismal predictors of postoperative survival. Furthermore, when the patients received adjuvant treatment, failure to complete all planned cycles reduced survival, as expected (HR = 4.45).

**Table 5:** Cox proportional hazard model to estimate the factors affecting patients’ survival following LDP.

	Hazard ratio	95% Confidence interval	p value
**Age, year**	0.932	0.891 to 0.975	0.002
**Size, cm**	1.311	1.002 to 1.714	0.049
**N stage**			
N0	Reference		
N1	3.113	1.330 to 7.286	0.009
**Combined resection**			
No	Reference		
Yes	4.503	1.215 to 16.697	0.024
**Planned adjuvant treatment**			
Complete	Reference		
Incomplete	4.448	1.847 to 10.713	0.001

## Discussion

Laparoscopic pancreatectomy has been accepted as a standard procedure for benign or low-grade malignant disease. Previous studies showed that laparoscopic pancreatectomy has similar surgical outcomes to open pancreatectomy [7–13,19–21]. Although the laparoscopic technique is not yet a commonly accepted approach for pancreatic cancer, several studies have reported the advantages and comparable outcomes of the laparoscopic approach [22–26]. We also reported, based on the extensive experience of our institute, that LDP for left-sided ductal adenocarcinoma showed several advantages over ODP and comparable oncologic outcomes [14]. In our present study, we have reported our experiences and appraised the value of LDP in all patients who underwent LDP for left-sided pancreatic cancer in our institute.

There are three clinical implications of our current study. First, the surgical outcomes of LDP were similar to those of ODP and LDP reported in previous articles in terms of oncologic outcomes and the quality of the procedure [22–29]. As summarized in Table 6, all studies show comparable surgical outcomes. In our present study, the clinically significant complication rate was 17.7% (Clavien-Dindo grade II-V) and the clinically relevant POPF rate (ISGPF grade B or C) was 9.2%. Previous studies [9,14,21,22,24,30] have reported similar results, and LDP did not compromise surgical outcomes.

**Table 6:** Summary of previously reported articles associated with operative outcomes of ODP and/or LDP for left-sided pancreatic cancer.

First author	Year (study years)	No. of cases	OP duration (min)	Tumor size (cm)	No. of harvested LN	N1 (%)	R0 (%)	Overall morbidity (%)	LOS (day)	Rate of adjuvant chemotherapy (%)	Median survival (mo)	Patient survival
**ODP**												
Yamamoto et al^29^	2010 (1994–2007)	73	(M) 345	(M) 2.8	NS	50	75.3	NS	NS	34.2	NS	5YSR 30.0%
Kooby et al^23^	2010 (2000–2008)	189	(m) 230.4	(m) 4.5	(m) 12.5	NS	73	NS	(m) 10.7	70	16	NS
Mitchem et al^27^	2012 (1999–2008)	47	(m) 243	(m) 4.4	(m) 18	55	81	83^a^	(m) 11.3	NS	26	5YSR 35.5%
Magge et al^24^	2013 (2002–2010)	34	(m) 294	(M) 4.5	(M) 12	38	88	50^b^	(M) 8	85	19	NS
Rehman et al^25^	2014 (2008–2011)	8	(M) 376	(M) 3.2	(M) 14	64	86	22 ^b^	(M) 12	64	52	NS
Paye et al^28^	2015 (2004–2009)	278	NS	NS	(M) 17	58.1	74.8	34.5 ^b^	NS	71.3	35	5YSR 29.5%
Sharpe et al^26^	2014 (2010–2011)	625	NS	(M) 3.6	(M) 12	49	78	NS	(M) 7	NS	NS	NS
**LDP**												
Kooby et al^23^	2010 (2000–2008)	23	(m) 238.4	(m) 3.5	(m) 13.8	NS	74	NS	(m) 7.4	57	16	NS
Magge et al^24^	2013 (2002–2010)	28	(m) 317	(M) 3.7	(M) 11	57	86	39 ^b^	(M) 6	89	19	NS
Rehman et al^25^	2014 (2008–2011)	14	(M) 274	(M) 2.2	(M) 16	50	88	37 ^b^	(M) 8	50	33	NS
Kawaguchi et al^22^	2014 (2002–2013)	23	(m) 203	(m) 3.2	(m) 20	61	100	47 ^b^	(m) 17	NS	28	5YSR 33.0%
Sharpe et al^26^	2014 (2010–2011)	144	NS	(M) 3.5	(M) 13	47	87	NS	(M) 5	NS	NS	NS
Current series	2015 (2006–2014)	152	(M) 234	(M) 3.0	(M) 11	42.8	82.9	40.1 ^b^	(M) 8	63.2	37 ^c^	5YSR 39.3%^c^

ODP, open distal pancreatectomy; LDP, laparoscopic distal pancreatectomy; LN, lymph node; LOS, length of stay; NS, not stated; 5YSR, 5 year survival rate; 3YSR, 3 year survival rate; (M), Median; (m), mean

^a^, based on the Revised Accordion Classification

^b^, based on the Clavien-Dindo Complication Classification (grade I-V)

^c^, estimated survival of 130 patients who underwent LDP for left-sided ductal adenocarcinoma

International Study Group on Pancreatic Surgery (ISGPS) reported a consensus statement for lymphadenectomy in surgery for PDAC [31]. In this report, they recommended proper extent and number of a standard lymphadenectomy in pancreatic surgery. For cancers of the body and tail of the pancreas, removal of stations 10 (LNs at the splenic hilum), 11 (LNs along the splenic artery) and 18 (LNs along the inferior border of the body and tail of the pancreas) was standard, but there was no guideline for proper number of LNs retrieved during distal pancreatectomy. Ashfaq et al [32] studied the number of LNs required for accurate staging after distal pancreatectomy for pancreatic adenocarcinoma, and reported that at least 11 LNs should be examined to avoid understaging. The median or mean number of harvested LNs of LDP indicated in Table 6 ranges from 11 to 20. Although the median or mean values are not an absolute standard, the quality of LDP can be considered oncologically feasible and comparable to that of ODP. Additionally, a negative surgical margin is one of the important prognostic factors for assessing oncologic adequacy. Although the RAMPS procedure was initially devised to achieve negative surgical margins and complete node dissections [18], the true oncologic and survival benefits have not achieved consensus [33,34]. Nevertheless, we believe that the concept of securing the retroperitoneal radial margin should be accepted, so we have generally followed it, sometimes modifying the procedure to reduce the time required. In this study, we achieved 82.9% R0 resection. Among the 25 patients who were categorized as having a positive retroperitoneal margin (R1), the cancer in 11 patients did not microscopically penetrate the retroperitoneal radial margin, but their safety margin was less than 1 mm (Table 3). In addition, we laparoscopically performed combined resection of invaded organs in 13 patients (8.6%). Yamamoto et al [29] reported that they performed combined resection of invaded organs, including the portal vein, in 20.5% patients during ODP. Although vascular resection and anastomosis is not an easy procedure in laparoscopic surgery, invasion to any other left-sided organs around the pancreas (stomach, colon, small intestine, and kidney) can be treated with appropriate laparoscopically obtainable surgical margins.

The second clinical implication of our current study is improved recovery, earlier return to ordinary life and subsequent possibility of improved survival of patients undergoing laparoscopic surgery. In our previous matched study [14] and other comparative studies [9,11,21,25,26], LDP vs ODP showed that LDP was associated with a shorter operative time, shorter length of hospital stay, earlier return to diet and earlier return to ordinary life. These associated characteristics of the laparoscopic procedure meant that improved recovery from surgery led to more patients receiving adjuvant treatment in a shorter period. Previous studies of colon cancer [35] and ovarian cancer [36] have suggested that delayed initiation of adjuvant chemotherapy compromised overall survival. In the recent analysis of the European Study Group for Pancreatic Cancer-3 (ESPAC-3) trial, Valle et al [37] showed that completion of all 6 cycles of planned adjuvant chemotherapy was more predictive of survival than early initiation if chemotherapy was initiated within 12 weeks. However, they did not evaluate the survival of patients whose chemotherapy initiation was delayed beyond 12 weeks. Croome et al [38] reported that an adjuvant chemotherapy delay beyond 90 days (12 weeks) was a strong predictor of a dismal prognosis and that a significantly smaller proportion of patients had a delay of greater than 90 days in the laparoscopic pancreaticoduodenectomy group. In our present study, 63.2% of patients received adjuvant treatment. The median time to adjuvant chemotherapy was 30 days after surgery (range, 19–70). Two patients had a delay of more than 8 weeks (61 and 70 days), but no patient had a delay longer than 12 weeks. Additionally, we expect that the improved recovery following the laparoscopic procedure could lead to more patients completing the planned adjuvant treatment. Of the patients receiving adjuvant treatment, 75% completed the planned chemotherapy cycle. When compared with the previous prospective trials that showed a 68% completion rate [37,39], more patients who completed all planned cycles in this study showed a higher probability of survival enhancement in the laparoscopic procedure. Our current data actually showed 5-year survival rates of the entire cohort (n = 152) and of patients with ductal adenocarcinoma (n = 130) of 44.7% and 39.3%, respectively. Acknowledging that patients who underwent LDP had relatively limited stages of cancer, we expected that this higher survival was attributable to not only tumor characteristics, but also to earlier recovery following laparoscopic procedure. Therefore, further study with high quality will be required to identify the correlation between early recovery and long-term oncologic outcomes in terms of receiving and completing the planned adjuvant treatment earlier.

As the last implication of this study, we suggest relative indications and contraindications of LDP for left-sided pancreatic cancer. All types and all stages of left-sided pancreatic cancer can be treated by the laparoscopic procedure if the cancer belongs to a resectable category, even if the tumor invades adjacent organs. The instances of conversion to open surgery in this study could provide useful information for establishing the contraindications to LDP. When left-sided pancreatic cancer invades major vessels, such as the portal vein, superior mesenteric vein, celiac axis, and superior mesenteric artery, these vessels should be preserved by reconstruction. Therefore, LDP is not suitable for patients with major vascular invasion. Moreover, when there are severe adhesions caused by previous abdominal surgery, the laparoscopic approach might not be a safe choice.

Our study has several limitations. There were selection biases caused by limitations in laparoscopic procedure. Although we reported in a previous comparative study [14] that there were no specific differences in the selection criteria in terms of clinicopathological parameters except for tumor size and concurrent resection of invaded organs, LDP had limitations in dealing with large sized cancer invading adjacent organs or locally advanced cancer. Relatively short follow-up duration of recent patients was also associated with another bias in calculating accurate survival. Additionally, although the median number of retrieved LN was similar with other studies and suggested statement, the median value showed that many patients had fewer than the recommended number of LNs. Among the studied patients, some patients with 0 or 1 of retrieved LN were preoperatively diagnosed to have premalignant disease. In these patients with incidental PDAC, additional operation for lymphadenectomy was not performed when resection margin was clear. This might be associated with a bias on accurate staging.

## Conclusions

Acknowledging that this was a retrospective study and that the follow-up duration was relatively short, we appraised that LDP for left-sided pancreatic cancer was feasible or beneficial in terms of oncologic aspects. We believe that this approach is thoroughly reasonable within the selected indications and that it is time to establish an international consensus on laparoscopic surgery for pancreatic cancer.

## Supporting Information

S1 File Data sheet for analysis. (PDF)
