# Supplementary material for: Appraisal of Laparoscopic Distal Pancreatectomy for Left-Sided Pancreatic Cancer: A Large Volume Cohort Study of 152 Consecutive Patients
Source: PLoS One. 2016 Sep 16;11(9):e0163266. doi: 10.1371/journal.pone.0163266 (PMC5026333; doi:10.1371/journal.pone.0163266)
Supplement: S1 File — (PDF) [file pone.0163266.s001.pdf]

ID	sex	age	ASA	BMI	POD	op time	DM
1	0	70	2	27.17	11	240	0
2	1	67	2	24.05	7	187	1
3	1	61	2	25.22	7	303	1
4	0	56	2	21.65	11	290	1
5	0	67	3	21.91	7	290	1
6	1	66	2	25	10	212	0
7	1	65	2	23.79	11	308	0
8	0	71	2	16.57	8	286	1
9	0	76	2	19.74	7	168	0
10	0	57	2	23.91	7	193	0
11	0	64	2	23.31	7	243	1
12	0	68	2	23.36	8	226	1
13	0	59	2	27.14	13	254	1
14	1	55	2	21.68	6	249	1
15	1	60	2	26.6	11	261	1
16	1	68	3	21.17	8	236	1
17	0	70	2	25.31	8	208	1
18	1	70	2	24.04	13	250	0
19	0	74	2	26.14	13	229	1
20	0	83	2	24.19	8	230	0
21	0	69	1	26.76	9	203	0
22	1	64	1	24.83	8	234	1
23	0	67	3	26.4	21	292	0
24	0	63	1	21.76	8	161	0
25	0	64	1	24.61	10	208	0
26	1	66	2	22.2	9	300	0
27	1	68	1	26.71	10	163	0
28	0	52	1	17.36	6	203	0
29	1	57	1	21.44	29	225	0
30	1	55	2	25.63	9	208	1
31	0	57	1	24.41	7	302	0
32	0	53	1	23.05	6	182	0
33	1	59	2	26.4	10	330	0
34	0	61	2	19.62	8	205	0
35	1	54	1	23.81	7	252	0
36	0	70	2	24.09	9	159	1
37	1	82	2	21.64	9	169	0
38	0	65	2	25.4	8	173	0
39	0	71	3	26.98	7	182	1
40	0	69	2	24.61	11	147	1
41	1	74	2	24.06	6	200	0
42	1	52	2	22.74	8	368	0

43	0	62	2	25.15	9	165	0
44	0	65	2	24.91	8	302	1
45	1	71	2	23.29	10	220	0
46	0	60	2	22.54	8	191	0
47	1	56	2	27.07	8	289	1
48	1	67	2	30.57	6	185	0
49	1	68	2	25.55	8	265	0
50	0	79	2	21.05	7	247	0
51	1	76	2	24.48	9	121	0
52	0	74	3	22.87	7	185	1
53	1	61	1	30.3	23	363	0
54	1	48	1	23.98	8	195	0
55	0	66	2	24.63	8	260	1
56	1	59	2	27.07	7	270	0
57	0	67	1	26.85	6	205	0
58	1	68	1	24.1	7	263	0
59	1	59	1	30.16	13	330	0
60	1	72	1	24.61	12	166	0
61	1	44	2	20.84	6	134	0
62	1	52	1	23.58	8	175	0
63	0	36	2	20.02	7	183	0
64	0	47	2	20.89	10	184	1
65	1	43	2	28.69	12	475	1
66	1	63	2	23.28	7	183	0
67	0	66	2	25.24	13	281	0
68	0	38	2	26.34	9	176	0
69	0	73	2	20.79	10	170	1
70	1	82	2	18.35	9	216	1
71	1	69	2	28.68	11	316	1
72	0	64	2	27.65	14	213	0
73	1	40	1	25.71	12	236	0
74	0	69	2	22.5	11	270	1
75	0	46	2	22.3	6	200	0
76	1	81	1	26.75	9	244	0
77	1	58	1	21.18	16	244	0
78	1	40	1	19.9	5	189	0
79	1	62	2	20.68	10	220	0
80	0	63	2	26.64	7	237	1
81	1	62	2	19.22	19	157	1
82	1	61	1	25.39	22	232	0
83	1	77	3	21.39	8	299	0
84	1	49	1	25.08	9	296	0
85	0	71	2	21.94	31	290	1

86	1	74	2	25.2	9	274	1
87	1	67	2	22.89	9	216	0
88	0	73	1	24.38	7	212	1
89	1	43	2	21.55	22	302	0
90	1	58	1	23.82	16	397	0
91	0	69	2	23.79	8	167	0
92	0	55	2	21.69	8	180	0
93	1	62	2	26.49	24	215	1
94	0	60	2	22.24	16	271	0
95	1	58	2	23.83	11	241	1
96	0	51	1	25.69	7	298	0
97	1	65	2	23.09	6	158	1
98	1	65	1	21.25	7	266	1
99	1	54	1	26.15	7	208	0
100	1	55	2	21.09	9	285	1
101	0	63	2	18.86	9	125	0
102	1	61	1	21.75	13	294	0
103	1	86	3	23.18	7	225	0
104	1	56	2	26.39	11	308	0
105	1	65	3	24.1	7	220	0
106	1	65	2	21.48	7	207	0
107	1	82	2	22.97	27	280	0
108	1	60	2	20.37	5	214	1
109	1	55	2	25.71	11	332	0
110	1	62	2	25.18	10	356	1
111	0	70	2	21.01	9	260	0
112	0	56	2	25.19	6	252	1
113	1	39	2	25.91	6	265	0
114	1	66	2	27.78	8	249	0
115	1	50	2	27	25	372	0
116	0	75	3	25.68	12	188	1
117	1	54	1	22.73	6	205	1
118	1	52	2	25	14	252	0
119	1	53	2	21.33	6	288	0
120	0	73	2	28.25	7	285	0
121	1	58	2	22.74	21	346	0
122	1	30	1	25.25	9	177	0
123	0	64	1	22.62	7	267	0
124	1	47	1	24.33	5	197	0
125	1	78	2	27.86	9	220	0
126	1	57	1	22.15	19	375	0
127	1	65	2	23.6	12	269	0
128	1	57	2	22.62	8	300	0

129	1	42	2	32.24	8	298	0
130	0	61	1	22.95	6	235	0
131	1	53	2	24.72	6	180	0
132	0	79	2	22.25	14	231	1
133	0	71	2	32.1	8	220	1
134	1	74	2	23.51	8	209	1
135	1	58	1	21.54	7	360	1
136	0	73	2	18.75	7	216	1
137	1	51	2	22.53	20	270	0
138	1	71	2	26.29	11	272	0
139	1	88	2	26.3	9	246	0
140	1	53	2	24.1	7	208	0
141	1	61	2	24.7	10	280	0
142	1	59	2	21.69	17	213	1
143	1	50	2	23.28	8	285	0
144	0	46	2	31.5	14	194	1
145	0	54	1	21.78	7	251	0
146	1	64	2	24.11	21	385	0
147	1	60	2	21.83	7	305	1
148	0	50	2	23.15	7	211	0
149	1	68	2	23.22	8	206	0
150	1	43	1	25.48	13	190	0
151	0	58	2	25.04	8	243	0
152	0	65	2	24.37	22	200	0

op_date	recur	recur_date	recur site	death	LFUD	PtS	DFS
15-Nov-13	0	01-Nov-15	0	0	01-Nov-15	23.9	23.9
26-Jun-13	0	01-Nov-15	0	0	01-Nov-15	28.6	28.6
14-Jan-11	0	01-Nov-15	0	0	01-Nov-15	58.4	58.4
05-Aug-10	0	01-Nov-15	0	0	01-Nov-15	63.8	63.8
02-Jan-14	0	01-Nov-15	0	0	01-Nov-15	22.3	22.3
16-Dec-13	0	01-Nov-15	0	0	01-Nov-15	22.8	22.8
20-Jun-13	0	01-Nov-15	0	0	01-Nov-15	28.8	28.8
31-Oct-14	0	01-Nov-15	0	0	01-Nov-15	12.2	12.2
28-Nov-14	0	01-Nov-15	0	0	01-Nov-15	11.3	11.3
11-Jun-14	0	01-Nov-15	0	0	01-Nov-15	16.9	16.9
26-Sep-13	0	01-Nov-15	0	0	01-Nov-15	25.5	25.5
09-Aug-12	0	01-Nov-15	0	0	01-Nov-15	39.3	39.3
21-Feb-13	0	01-Nov-15	0	0	01-Nov-15	32.8	32.8
19-Jul-12	0	01-Nov-15	0	0	01-Nov-15	40	40
01-Dec-11	0	01-Nov-15	0	0	01-Nov-15	47.7	47.7
03-May-11	0	01-Nov-15	0	0	01-Nov-15	54.8	54.8
16-Mar-11	0	01-Nov-15	0	0	01-Nov-15	56.4	56.4
30-Dec-10	0	01-Nov-15	0	0	01-Nov-15	58.9	58.9
23-Apr-13	0	01-Nov-15	0	0	01-Nov-15	30.7	30.7
22-Nov-12	0	01-Nov-15	0	0	01-Nov-15	35.8	35.8
18-Apr-12	0	01-Nov-15	0	0	01-Nov-15	43.1	43.1
15-May-12	0	01-Nov-15	0	0	01-Nov-15	42.2	42.2
22-Jun-11	0	01-Nov-15	0	0	01-Nov-15	53.1	53.1
02-Jul-13	0	01-Nov-15	0	0	01-Nov-15	28.4	28.4
08-Mar-13	0	01-Nov-15	0	0	01-Nov-15	32.3	32.3
24-Nov-11	0	01-Nov-15	0	0	01-Nov-15	47.9	47.9
04-May-10	0	01-Nov-15	0	0	01-Nov-15	66.9	66.9
25-Apr-12	0	01-Nov-15	0	0	01-Nov-15	42.8	42.8
07-Jun-11	0	01-Nov-15	0	0	01-Nov-15	53.6	53.6
20-Nov-14	0	01-Nov-15	0	0	01-Nov-15	11.5	11.5
25-Nov-14	0	01-Nov-15	0	0	01-Nov-15	11.4	11.4
06-Aug-14	0	01-Nov-15	0	0	01-Nov-15	15.1	15.1
16-Apr-14	0	01-Nov-15	0	0	01-Nov-15	18.8	18.8
09-Apr-14	0	01-Nov-15	0	0	01-Nov-15	19	19
25-Mar-13	0	01-Nov-15	0	0	01-Nov-15	31.7	31.7
15-Sep-14	0	01-Nov-15	0	0	01-Nov-15	13.7	13.7
16-May-07	0	01-Nov-15	0	0	01-Nov-15	103	103
22-Aug-14	0	01-Nov-15	0	0	01-Nov-15	14.5	14.5
01-Jul-14	0	01-Nov-15	0	0	01-Nov-15	16.3	16.3
17-Sep-13	0	01-Nov-15	0	0	01-Nov-15	25.8	25.8
14-Jun-13	0	01-Nov-15	0	0	01-Nov-15	29	29
05-Apr-12	0	01-Nov-15	0	0	01-Nov-15	43.5	43.5

14-Mar-12	0	01-Nov-15	0	0	01-Nov-15	44.2	44.2
22-Dec-14	0	01-Nov-15	0	0	01-Nov-15	10.5	10.5
21-Jul-14	0	01-Nov-15	0	0	01-Nov-15	15.6	15.6
13-Feb-14	0	01-Nov-15	0	0	01-Nov-15	20.9	20.9
05-Mar-13	0	01-Nov-15	0	0	01-Nov-15	32.4	32.4
19-Jun-07	0	01-Nov-15	0	0	01-Nov-15	101.9	101.9
16-Dec-14	0	01-Nov-15	0	0	01-Nov-15	10.7	10.7
24-Jun-14	0	01-Nov-15	0	0	01-Nov-15	16.5	16.5
28-Nov-14	0	01-Nov-15	0	0	01-Nov-15	11.3	11.3
12-Sep-13	0	01-Nov-15	0	0	01-Nov-15	26	26
11-Mar-13	0	01-Nov-15	0	0	01-Nov-15	32.2	32.2
03-Jun-14	0	01-Nov-15	0	0	01-Nov-15	17.2	17.2
17-Dec-14	0	01-Nov-15	0	0	01-Nov-15	10.6	10.6
10-Aug-12	0	01-Nov-15	0	0	01-Nov-15	39.3	39.3
20-Apr-10	0	01-Nov-15	0	0	01-Nov-15	67.4	67.4
04-Apr-13	0	01-Nov-15	0	0	01-Nov-15	31.4	31.4
23-Sep-09	0	01-Nov-15	0	0	01-Nov-15	74.3	74.3
11-Dec-06	0	01-Nov-15	0	0	01-Nov-15	108.2	108.2
12-Apr-13	0	01-Nov-15	0	0	01-Nov-15	31.1	31.1
03-Jun-08	0	01-Nov-15	0	0	01-Nov-15	90.2	90.2
22-Jan-14	0	01-Nov-15	0	0	01-Nov-15	21.6	21.6
19-Aug-13	0	01-Nov-15	0	0	01-Nov-15	26.8	26.8
08-Nov-12	0	01-Nov-15	0	0	01-Nov-15	36.3	36.3
15-Dec-11	0	01-Nov-15	0	0	01-Nov-15	47.2	47.2
01-Jul-08	0	01-Nov-15	0	0	01-Nov-15	89.3	89.3
12-Jun-07	0	01-Nov-15	0	0	01-Nov-15	102.1	102.1
17-Sep-14	0	01-Nov-15	0	0	01-Nov-15	13.7	13.7
21-Jan-14	0	01-Nov-15	0	0	01-Nov-15	21.6	21.6
16-Sep-14	0	01-Nov-15	0	0	01-Nov-15	13.7	13.7
26-Nov-13	0	01-Nov-15	0	0	01-Nov-15	23.5	23.5
15-Jan-13	0	01-Nov-15	0	0	01-Nov-15	34	34
25-Mar-10	0	01-Nov-15	0	0	01-Nov-15	68.2	68.2
13-Jan-10	0	01-Nov-15	0	0	01-Nov-15	70.6	70.6
12-Jul-13				1	03-Jun-15	23	
13-Sep-07	1	15-Oct-08	2	1	16-Mar-10	30.5	13.3
11-Dec-09	1	05-Feb-10	2	1	10-May-10	5	1.9
18-Nov-09	1	30-Apr-10	2	1	13-Oct-12	35.3	5.4
24-Apr-09	1	27-Aug-10	1	1	23-Jun-11	26.3	16.3
12-Nov-09	1	07-Oct-10	2	1	30-Jun-12	32	11
03-Jun-10	1	13-Oct-10	2	1	13-Jul-11	13.5	4.4
04-Aug-10	1	25-Nov-10	2	1	23-Mar-11	7.7	3.8
04-Oct-10	1	29-Apr-11	2	1	03-Sep-13	35.5	6.9
08-Sep-10	1	02-May-11	2	1	20-Apr-14	44	7.9

18-May-11	1	26-May-11	2	1	03-Sep-11	3.6	0.3
22-Mar-11	1	26-Aug-11	2	1	27-Jul-12	16.4	5.2
09-May-11	1	12-Oct-11	2	1	21-Apr-12	11.6	5.2
08-Dec-10	1	04-Jan-12	2	1	05-Jul-13	31.3	13.1
31-Dec-10	1	16-Feb-12	1	1	04-Dec-14	47.8	13.7
02-Dec-11	1	22-Mar-12	2	1	22-Nov-12	11.9	3.7
01-Sep-11	1	06-Apr-12	2	1	22-Jun-13	22	7.3
20-Mar-12	1	12-Jun-12	2	1	30-Jun-12	3.4	2.8
01-Feb-11	1	26-Jun-12	1	1	29-Oct-13	33.4	17
20-Dec-11	1	04-Jul-12	2	1	30-Sep-13	21.7	6.6
06-Oct-10	1	17-Aug-12	2	1	09-Jan-14	39.7	22.7
25-Jun-09	1	05-Sep-12	2	1	06-Jan-13	43	38.9
25-Nov-11	1	27-Sep-12	1	1	01-Jul-13	19.5	10.2
10-Feb-12	1	10-Oct-12	1	1	29-Dec-12	10.8	8.1
18-Nov-11	1	12-Oct-12	2	1	23-Dec-13	25.5	11
07-Dec-07	1	05-Dec-12	2	0	01-Nov-15	96.2	60.8
13-Jun-12	1	17-Apr-13	2	1	01-Sep-13	14.8	10.3
23-May-12	1	24-Apr-13	2	1	24-Apr-15	35.5	11.2
21-Jun-12	1	25-Apr-13	2	1	06-Oct-13	15.7	10.3
10-Jan-13	1	29-Apr-13	2	1	08-Jan-14	12.1	3.6
06-Dec-12	1	24-May-13	2	1	17-Apr-14	16.6	5.6
18-Sep-12	1	28-May-13	1	1	18-Sep-13	12.2	8.4
18-May-12	1	31-May-13	2	1	03-Aug-13	14.7	12.6
22-Mar-12	1	07-Jun-13	2	1	24-May-15	38.6	14.7
04-Oct-12	1	24-Jul-13	1	1	12-Oct-14	24.6	9.8
08-Apr-13	1	27-Jul-13	2	1	19-May-14	13.5	3.7
26-Jan-12	1	29-Jul-13	2	1	23-Dec-13	23.2	18.3
06-Nov-12	1	26-Aug-13	2	1	04-Jul-14	20.2	9.8
20-Jan-10	1	01-Sep-13	2	1	17-Oct-13	45.5	44
25-Jul-13	1	20-Sep-13	2	1	19-Dec-13	4.9	1.9
02-Oct-12	1	10-Oct-13	2	1	01-Dec-13	14.2	12.4
27-Mar-13	1	25-Oct-13	1	1	18-Jul-14	15.9	7.1
08-Feb-13	1	17-Dec-13	2	1	27-Jun-14	16.8	10.4
22-Jun-12	1	26-Dec-13	2	1	08-Jul-15	37	18.4
10-May-12	1	16-Jan-14	1	1	03-Nov-14	30.2	20.5
27-Sep-13	1	23-Jan-14	2	1	04-Jul-14	9.3	3.9
16-Dec-13	1	28-Mar-14	2	1	25-Sep-14	9.4	3.4
30-Jan-13	1	28-Mar-14	2	1	24-Apr-15	27.1	14.1
11-Mar-13	1	01-Apr-14	2	1	27-Jun-14	15.8	12.9
16-Sep-13	1	30-Apr-14	2	1	05-Nov-14	13.8	7.5
09-Aug-13	1	30-Apr-14	2	1	30-Dec-14	16.9	8.8
28-Oct-13	1	12-Jun-14	2	1	18-Feb-15	15.9	7.6
23-Oct-13	1	13-Jun-14	2	0	01-Nov-15	24.6	7.8

04-Dec-13	1	06-Aug-14	1	1	11-May-15	17.4	8.2
12-Jun-12	1	27-Aug-14	1	0	01-Nov-15	41.2	26.9
03-Feb-14	1	13-Sep-14	2	1	10-Apr-15	14.4	7.4
10-Mar-14	1	19-Sep-14	2	1	11-May-15	14.2	6.4
13-Apr-10	1	01-Oct-14	1	0	01-Nov-15	67.6	54.4
04-Apr-14	1	02-Oct-14	2	1	30-Oct-14	7	6
21-Aug-13	1	06-Oct-14	2	1	24-May-15	21.4	13.7
02-Jul-14	1	04-Nov-14	1	1	07-Jul-15	12.3	4.2
10-Dec-13	1	14-Nov-14	2	1	28-May-15	17.8	11.3
19-Jun-14	1	29-Nov-14	2	0	01-Nov-15	16.7	5.4
12-Dec-13	1	01-Dec-14	2	0	01-Nov-15	23	11.8
17-Jul-13	1	09-Dec-14	2	1	24-Jun-15	23.6	17
17-Sep-13	1	19-Dec-14	2	0	01-Nov-15	25.8	15.3
11-Sep-14	1	31-Dec-14	2	0	01-Nov-15	13.9	3.7
27-Aug-14	1	23-Jan-15	2	0	01-Nov-15	14.4	5
25-Jul-14	1	08-Mar-15	2	0	01-Nov-15	15.5	7.5
30-Sep-14	1	20-Mar-15	2	1	01-Jun-15	8.1	5.7
02-May-14	1	23-Mar-15	1	0	01-Nov-15	18.3	10.8
14-Oct-14	1	15-May-15	2	0	01-Nov-15	12.8	7.1
20-Feb-14	1	21-May-15	2	1	30-Aug-15	18.5	15.2
21-Nov-14	1	13-Jun-15	1	0	01-Nov-15	11.5	6.8
31-Jan-13	1	05-Aug-15	2	0	01-Nov-15	33.5	30.5
20-Apr-10	1	15-Oct-15	1	0	01-Nov-15	67.4	66.8
03-Jun-11	0	01-Nov-15	0	0	01-Nov-15	53.7	53.7

Neoadjuva	CTx	RTx	adjuvant_day	until_chem	conversion	Coop	transfusior
0	1	0	10-Dec-13	25	0	0	1
0	1	0	17-Jul-13	21	0	0	0
0	1	0	08-Feb-11	25	0	0	0
0	1	0	03-Sep-10	29	0	0	0
0	0	0			0	0	0
0	0	0			0	1	0
0	0	0			0	0	0
0	1	0	05-Dec-14	35	0	0	0
0	0	0			0	0	0
0	0	0			0	0	0
0	1	0	23-Oct-13	27	0	0	0
0	0	0			0	0	0
0	1	0	20-Mar-13	27	0	0	0
0	1	0	14-Aug-12	26	0	0	0
0	1	1	03-Jan-12	33	0	0	0
0	1	1	31-May-11	28	0	0	0
0	1	0	14-Apr-11	29	0	0	0
0	1	0	26-Jan-11	27	0	0	0
0	0	0			0	0	0
0	0	0			0	0	0
0	0	0			0	0	0
0	1	1	29-Jun-12	45	0	0	0
0	0	0			0	0	0
0	1	0	31-Jul-13	29	0	0	0
0	1	0	01-Apr-13	24	0	0	0
0	1	1	23-Dec-11	29	0	0	1
0	1	0	04-Jun-10	31	0	0	0
0	0	0			0	0	0
0	0	0			0	0	0
0	1	0	01-Jan-15	42	0	1	0
0	1	0	24-Dec-14	29	0	0	0
0	1	0	03-Sep-14	28	0	0	0
0	1	0	26-May-14	40	0	0	0
0	1	0	14-May-14	35	0	0	0
0	1	0	17-Apr-13	23	0	0	0
0	0	0			0	0	0
0	0	0			0	0	0
0	1	0	29-Sep-14	38	0	0	0
0	1	0	01-Aug-14	31	0	0	0
0	1	0	21-Oct-13	34	0	0	0
0	1	0	08-Aug-13	55	0	0	0
0	1	0	25-Apr-12	20	0	0	0

0	1	0	25-Apr-12	42	0	0	0
0	0	0			0	0	0
0	0	0			0	0	0
0	0	0			0	0	0
0	0	0			0	0	0
0	0	0			0	0	0
0	1	0	19-Jan-15	34	0	0	0
0	0	0			0	0	0
0	0	0			0	0	0
0	1	0	01-Oct-13	19	0	0	0
0	0	0			0	0	0
0	0	0			0	0	0
0	1	0	29-Jan-15	43	0	0	0
0	1	0	05-Sep-12	26	0	0	0
0	0	0			0	0	0
0	1	0	15-May-13	41	0	1	0
0	1	0	21-Oct-09	28	0	0	0
0	1	0	15-Jan-07	35	0	0	0
0	0	0			0	0	0
0	1	0			0	0	0
0	1	0	12-Feb-14	21	0	0	0
0	1	0	26-Sep-13	38	0	0	0
0	1	0	05-Dec-12	27	0	0	0
0	1	1	20-Jan-12	36	0	0	0
0	1	0	04-Aug-08	34	0	0	0
0	1	0	01-Jul-07	19	0	0	0
0	0	0			0	0	0
0	0	0			0	0	0
0	0	0			0	0	0
0	0	0			0	0	0
0	0	0			0	0	0
0	0	0			0	1	0
0	0	0			0	0	0
0	0	0			0	0	0
0	1	0	15-Oct-07	32	0	0	0
0	1	0	07-Jan-10	27	0	0	0
0	1	1	24-Dec-09	36	0	0	0
0	1	0	15-May-09	21	0	0	0
0	1	0	12-Jan-10	61	0	0	0
0	1	0	13-Jul-10	40	0	0	0
0	0	0			0	0	0
0	1	0	03-Nov-10	30	0	0	0
0	0	0			0	0	0

0	0	0			0	1	0
0	1	1	14-May-11	53	0	0	0
0	0	0			0	0	0
0	1	0			0	0	0
0	1	0	08-Feb-11	39	0	0	0
0	0	0			0	1	0
0	1	1	23-Sep-11	22	0	0	0
0	0	0			0	0	0
0	1	0	08-Mar-11	35	0	0	0
0	1	0	26-Jan-12	37	0	0	0
0	1	0	03-Nov-10	28	0	0	0
0	0	0			0	0	0
0	1	0	28-Dec-11	33	0	0	1
0	1	0	06-Mar-12	25	0	0	0
0	1	1	04-Jan-12	47	0	0	0
0	1	0	11-Jan-08	35	0	0	0
0	1	0	23-Jul-12	40	0	0	0
0	0	0			0	0	0
1	1	0	16-Jul-12	25	0	0	0
0	1	0	30-Jan-13	20	0	0	0
0	1	0	31-Dec-12	25	0	1	0
0	0	0			0	0	1
0	1	1	20-Jun-12	33	0	0	0
0	1	1	26-Apr-12	35	0	0	0
0	1	0	08-Nov-12	35	0	0	0
0	0	0			0	0	0
0	0	0			0	0	0
0	1	0	05-Dec-12	29	0	0	0
0	1	0	17-Feb-10	28	0	0	0
0	1	0	10-Sep-13	47	0	1	0
0	0	0			0	0	0
0	1	0	16-May-13	50	0	0	0
0	1	0	20-Mar-13	40	0	0	0
0	1	0	31-Aug-12	70	0	0	0
0	1	1	04-Jun-12	25	0	0	0
0	1	1	05-Nov-13	39	0	1	0
0	1	0	04-Feb-14	50	0	0	0
0	0	0			0	0	0
0	0	0			0	0	0
0	0	0			0	0	0
0	0	0			0	0	0
0	1	0	04-Dec-13	37	0	1	0
0	1	0	28-Nov-13	36	0	0	0

0	1	0	31-Dec-13	27	0	0	0
0	1	0	01-Aug-12	50	0	0	0
0	1	0	11-Mar-14	36	0	0	0
0	0	0			0	1	0
0	1	0	06-May-10	23	0	0	0
0	0	0			0	0	0
0	1	0	30-Sep-13	40	0	0	0
0	0	0			0	0	0
0	1	0	29-Jan-14	50	0	0	0
0	0	0			0	0	0
0	1	1	08-Jan-14	27	0	0	0
0	1	0	26-Aug-13	40	0	0	0
0	1	0	14-Oct-13	27	0	1	0
0	1	0	20-Oct-14	39	0	1	0
0	1	0			0	1	0
0	1	0	26-Aug-14	32	0	0	0
0	1	1	05-Nov-14	36	0	0	0
0	1	0	18-Jun-14	47	0	0	0
0	1	0	20-Nov-14	37	0	0	0
0	1	0	27-Mar-14	35	0	0	0
0	0	0			0	0	0
0	1	0	27-Feb-13	27	0	0	0
0	1	0	19-May-10	29	0	0	0
0	0	0			0	0	0

Transfusioı	RM	size	Differ	LN_harvest	stage	Tstage	Nstage
2	0	2	1	5	2.1	3	0
0	0	3.7	3	4	2.1	3	0
0	0	2.5	2	2	2.1	3	0
0	0	2.5		7	2.2	3	1
0	0	2.5	2	18	2.1	3	0
0	0	3.5	2	18	2.2	3	1
0	0	2.5	2	6	2.1	3	0
0	1	2.9	2	16	2.2	3	1
0	1	2.7	2	10	2.1	3	0
0	0	3.5	2	14	2.1	3	0
0	1	5	2	11	2.2	3	1
0	0	2		9	1.1	1	0
0	0	2.5	2	15	2.1	3	0
0	0	5.6	2	15	2.2	3	1
0	1	3.8	2	28	2.1	3	0
0	1	2.7	2	7	2.2	3	1
0	0	3.5	2	10	2.2	3	1
0	1	3	1	22	2.2	3	1
0	1	2.2	1	13	2.1	3	0
0	0	2.5	2	14	2.2	3	1
0	0	2.5	2	10	2.1	3	0
0	1	3.5	2	5	2.2	3	1
0	0	2.2	2	14	2.1	3	0
0	0	3	2	15	2.1	3	0
0	0	2	1	11	2.1	3	0
2	0	3.1	2	31	2.2	3	1
0	0	2.4	2	0	2.1	3	0
0	0	1.5	2	21	2.1	3	0
0	0	3.5	2	10	2.2	3	1
0	0	2.9	2	18	2.2	3	1
0	0	1.5	2	14	2.1	3	0
0	1	2.8	2	21	2.2	3	1
0	0	3	2	7	2.1	3	0
0	0	2.8	3	14	2.2	3	1
0	0	2.5	2	36	2.1	3	0
0	0	2.8	2	14	2.1	3	0
0	0	2		0	1.2	2	0
0	0	3.3	3	7	2.2	3	1
0	0	1.5	1	6	2.1	3	0
0	0	6.3	3	10	2.2	3	1
0	0	2.5	2	6	2.2	3	1
0	0	5.5		7	2.1	3	0

0	0	0.9		3	1.1	1	0
0	1	3.1	2	6	2.1	3	0
0	0	2	1	3	2.1	3	0
0	0	1.8	1	0	1.1	1	0
0	0	3	1	1	2.1	3	0
0	0	2		0	1.2	2	0
0	0	2.5	1	14	2.2	3	1
0	1	3.4	2	16	2.2	3	1
0	0	1.6	2	9	1.2	2	0
0	0	2.8	2	7	2.1	3	0
0	1	1.5		3	2.1	3	0
0	0	1.8	1	12	1.1	1	0
0	0	2.2	1	18	1.2	2	0
0	0	3.5	2	3	2.1	3	0
0	0	5.5	1	3	1.2	2	0
0	0	5.8	1	10	2.1	3	0
0	0	2.5	2	6	2.1	3	0
0	0	1.8	2	13	2.2	3	1
0	0	0.4	1	0	2.1	3	0
0	0	3.5	1	1	2.1	3	0
0	0	2.3	2	15	2.2	3	1
0	0	1.1		0	1.1	1	0
0	0	2.5	2	3	2.1	3	0
0	0	2		4	2.1	3	0
0	0	2	1	17	2.1	3	0
0	0	8		0	1.2	2	0
0	0	7		10	2.1	3	0
0	1	0.8	1	3	2.1	3	0
0	0	5.6		5	2.1	3	0
0	0	0.6	1	0	1.1	1	0
0	0	0.4		1	1.1	1	0
0	0	1.5		0	1.1	1	0
0	0	2.5		0	1.2	2	0
0	1	3	2	7	2.2	3	1
0	0	1.9	2	2	2.1	3	0
0	1	3	2	5	2.1	3	0
0	1	4	2	9	2.2	3	1
0	0	0.9		0	2.1	3	0
0	0	2.5	2	0	2.1	3	0
0	0	1.8	2	23	2.2	3	1
0	0	10	2	5	2.1	3	0
0	0	4.5	2	6	2.1	3	0
0	0	1.5	2	3	2.2	1	1

0	1	5	2	9	2.2	3	1
0	1	5	2	20	2.2	3	1
0	0	4	2	21	2.1	3	0
0	0	6	1	20	2.2	3	1
0	0	6.5	2	8	2.1	3	0
0	0	4	2	7	2.1	3	0
0	0	3.1	3	9	2.2	3	1
0	0	3.3	3	7	2.1	3	0
0	0	3	2	12	2.2	3	1
0	0	2.6	1	27	2.2	3	1
0	0	1.8	2	34	2.2	3	1
0	0	0.5		3	1.1	1	0
2	0	5	2	17	2.2	3	1
0	0	1.8	3	11	2.1	3	0
0	0	2.7	1	29	2.2	3	1
0	0	1.5	2	9	2.1	3	0
0	1	3.2	2	17	2.2	3	1
0	0	3.2	2	12	2.1	3	0
0	0	3	2	14	2.2	3	1
0	0	5.5	2	9	2.2	3	1
0	0	3	2	10	2.2	3	1
2	0	3	2	7	2.1	3	0
0	1	8.5	2	9	2.2	3	1
0	0	3.3	2	20	2.2	3	1
0	0	6.5	2	15	2.2	3	1
0	0	1.8	2	8	2.1	3	0
0	0	3.7	2	26	2.2	3	1
0	0	4.5	2	21	2.2	3	1
0	0	2.7	3	16	2.2	3	1
0	1	3.7	2	7	2.2	3	1
0	0	1.2	2	34	1.1	1	0
0	0	3.5	2	8	2.1	3	0
0	0	1.8	2	9	1.1	1	0
0	0	4.5	2	30	2.2	3	1
0	1	4	2	19	2.2	3	1
0	1	6	2	21	2.2	3	1
0	0	2.1	2	18	2.1	3	0
0	0	5	2	4	2.2	3	1
0	0	4.2	2	26	2.2	3	1
0	1	2.6	2	7	2.2	3	1
0	1	5.5	2	20	2.2	3	1
0	0	3.3	2	22	2.2	3	1
0	0	6.6	3	42	2.2	3	1

0	0	5.5	2	7	2.2	3	1
0	0	2.5	2	26	2.1	3	0
0	1	5.2	2	11	2.1	3	0
0	0	6	2	6	2.1	3	0
0	0	4.5		8	1.2	2	0
0	0	3.7	2	19	2.2	3	1
0	0	4.5	2	9	2.2	3	1
0	0	1.8	2	13	2.1	3	0
0	0	2	2	7	2.2	3	1
0	0	3.5	2	10	2.1	3	0
0	0	2.5	2	12	2.1	3	0
0	0	1	1	12	2.2	3	1
0	0	6	2	14	2.1	3	0
0	0	5.3	2	5	2.1	3	0
0	0	3.2	3	2	2.1	3	0
0	0	7	2	4	2.2	3	1
0	1	4	2	4	2.2	3	1
0	0	4.7	2	15	2.1	3	0
0	0	6.5	2	10	2.2	3	1
0	0	2.9	2	5	2.2	3	1
0	0	1.4	2	5	2.1	3	0
0	0	2.2	3	18	2.2	3	1
0	0	1.4	2	6	2.1	3	0
0	0	2.5	2	12	2.1	3	0

Mstage	LVi	PNi	POPF	PFGr	POPF_gr	Cx	Clavien
0	0	0	0	0	0	1	2
0	1	1	1	1	0	1	1
0	0	0	1	1	0	1	1
0	0	1	0	0	0	0	0
0	0	1	0	0	0	1	1
0	0	1	0	0	0	0	0
0	1	1	1	1	0	1	1
0	0	1	0	0	0	1	1
0	0	0	0	0	0	0	0
0	1	1	0	0	0	0	0
0	0	1	1	1	0	1	1
0	0	1	0	0	0	0	0
0	0	0	1	1	0	1	2
0	0	1	0	0	0	0	0
0	0	1	0	0	0	0	0
0	0	1	0	0	0	0	0
0	0	1	0	0	0	0	0
0	0	1	0	0	0	1	1
0	0	1	0	0	0	0	0
0	0	1	0	0	0	0	0
0	1	1	0	0	0	0	0
0	1	1	0	0	0	0	0
0	0	1	1	3	1	1	2
0	0	0	0	0	0	0	0
0	1	1	0	0	0	0	0
0	0	1	0	0	0	0	0
0	0	1	0	0	0	0	0
0	0	1	0	0	0	0	0
0	1	1	1	3	1	1	3
0	0	0	1	1	0	1	0
0	1	1	0	0	0	0	0
0	1	1	1	1	0	1	1
0	0	1	1	2	1	1	2
0	0	1	0	0	0	0	0
0	0	1	0	0	0	0	0
0	1	1	0	0	0	0	0
0	0	0	0	0	0	0	0
0	0	1	1	1	0	1	1
0	0	0	0	0	0	0	0
0	0	1	0	0	0	0	0
0	0	1	0	0	0	0	0
0	0	0	0	0	0	0	0

0	0	1	0	0	0	0	0
0	1	1	0	0	0	0	0
0	1	1	1	1	0	1	1
0	0	0	0	0	0	0	0
0	0	1	1	1	0	1	1
0	0	0	0	0	0	0	0
0	0	1	0	0	0	0	0
0	1	1	1	1	0	1	1
0	0	0	0	0	0	1	1
0	0	1	1	2	1	1	2
0	0	1	1	3	1	1	3
0	0	0	1	1	0	1	1
0	0	0	1	1	0	1	1
0	0	0	0	0	0	0	0
0	0	0	0	0	0	0	0
0	0	1	0	0	0	0	0
0	0	1	0	0	0	0	0
0	0	0	0	0	0	0	0
0	0	0	0	0	0	0	0
0	0	0	0	0	0	0	0
0	0	1	0	0	0	1	1
0	1	0	1	1	0	1	1
0	0	0	1	1	0	1	1
0	0	1	1	1	0	1	1
0	0	0	1	1	0	1	1
0	0	0	1	1	0	1	1
0	1	1	0	0	0	0	0
0	0	0	0	0	0	0	0
0	0	0	1	1	0	1	1
0	0	0	1	1	0	1	1
0	0	0	0	0	0	1	3
0	0	0	0	0	0	0	0
0	0	0	1	1	0	1	1
0	1	1	0	0	0	0	0
0	0	1	0	0	0	0	0
0	0	0	0	0	0	0	0
0	0	0	0	0	0	0	0
0	0	1	1	1	0	1	1
0	0	1	0	0	0	1	3
0	1	1	1	1	0	1	2
0	1	0	0	0	0	0	0
0	0	0	0	0	0	0	0
0	0	0	0	0	0	1	2

0	0	1	0	0	0	0	0
0	1	1	0	0	0	0	0
0	1	1	0	0	0	0	0
0	1	1	1	1	0	1	3
0	0	1	0	0	0	0	0
0	0	1	0	0	0	0	0
0	1	1	0	0	0	0	0
0	0	0	0	0	0	1	2
0	0	1	0	0	0	0	0
0	1	1	0	0	0	0	0
0	1	1	0	0	0	0	0
0	0	0	0	0	0	0	0
0	0	1	0	0	0	0	0
0	1	1	0	0	0	0	0
0	0	1	0	0	0	0	1
0	0	0	0	0	0	0	0
0	0	1	0	0	0	1	1
0	1	1	0	0	0	0	0
0	0	0	0	0	0	0	0
0	0	1	0	0	0	0	0
0	1	1	0	0	0	0	0
0	0	0	1	3	1	1	3
0	0	1	0	0	0	0	0
0	0	1	1	1	0	1	1
0	0	1	0	0	0	0	0
0	0	1	0	0	0	0	0
0	1	1	0	0	0	0	0
0	0	1	0	0	0	0	0
0	0	1	1	1	0	1	2
0	1	1	1	2	1	0	2
0	0	0	0	0	0	0	0
0	0	1	1	1	0	1	2
0	0	0	1	3	1	1	3
0	1	1	1	2	1	1	3
0	0	1	0	0	0	0	0
0	0	1	0	0	0	1	2
0	0	1	0	0	0	0	0
0	1	1	0	0	0	0	0
0	1	0	0	0	0	0	0
0	1	1	0	0	0	0	0
0	1	1	1	2	1	0	2
0	1	1	0	0	0	0	0
0	1	1	0	0	0	0	0

0	0	1	1	1	0	1	1
0	0	1	0	0	0	0	0
0	1	0	0	0	0	0	0
0	1	0	0	0	0	1	2
0	0	1	1	1	0	1	1
0	0	1	0	0	0	0	0
0	1	1	0	0	0	0	0
0	1	0	0	0	0	0	0
0	1	1	1	2	1	1	2
0	0	1	1	2	1	1	2
0	0	0	0	0	0	0	0
0	0	1	0	0	0	0	0
0	0	1	0	0	0	0	0
0	0	0	1	1	0	1	2
0	0	1	1	1	0	0	0
0	0	1	1	2	1	1	2
0	1	1	1	1	0	1	1
0	0	1	1	2	1	1	3
0	1	1	1	1	0	1	1
0	0	1	0	0	0	0	0
0	1	1	0	0	0	0	0
0	1	1	0	0	0	1	1
0	0	1	0	0	0	0	0
0	0	1	1	1	0	1	1

clavien_gr	diet
1	1
0	1
0	1
0	1
0	1
0	3
0	1
0	1
0	1
0	1
0	1
0	1
1	1
0	1
0	1
0	1
0	1
0	1
0	1
0	1
0	1
0	1
1	2
0	1
0	1
0	4
0	1
0	1
1	1
0	1
0	1
0	1
1	1
0	1
0	1
0	1
0	2
0	1
0	1
0	1
0	1
0	1

0	1
0	1
0	1
0	1
0	1
0	2
0	1
0	1
0	1
1	1
1	4
0	1
0	1
0	2
0	1
0	1
0	2
0	4
0	1
0	2
0	1
0	1
0	1
0	1
0	1
0	2
0	1
0	1
0	2
0	1
1	1
0	1
0	3
0	4
0	6
0	1
0	4
0	2
1	2
1	1
0	1
0	1
1	1

0	4
0	1
0	1
1	4
0	3
0	1
0	1
1	1
0	1
0	1
0	1
0	1
0	3
0	1
0	1
0	4
0	1
0	1
0	1
0	1
0	1
1	1
0	1
0	1
0	3
0	1
0	1
0	1
1	2
1	5
0	1
0	1
1	3
1	1
0	1
1	3
0	1
0	1
0	1
0	3
1	2
0	1
0	1

0	1
0	1
0	1
1	5
0	4
0	1
0	1
0	1
1	1
1	1
0	1
0	1
0	1
1	2
0	3
1	1
0	1
1	1
0	1
0	1
0	1
0	1
0	1
0	4
